# Hearing Results after Transmastoid Superior Semicircular Canal Plugging for Superior Semicircular Canal Dehiscence: A Meta-Analysis

**DOI:** 10.3390/audiolres13050065

**Published:** 2023-10-08

**Authors:** Efterpi Michailidou, Pascal Oliver Rüegg, Tanya Karrer, Athanasia Korda, Stefan Weder, Martin Kompis, Marco Caversaccio, Georgios Mantokoudis

**Affiliations:** 1 Department of Otorhinolaryngology, Head and Neck Surgery, Inselspital, University Hospital Bern, University of Bern, 3010 Bern, Switzerland; 2 Medical Library, University Library of Bern, University of Bern, 3010 Bern, Switzerland

**Keywords:** superior canal dehiscence syndrome, transmastoid, plugging, hearing loss

## Abstract

Objective: The transmastoid plugging of a superior semicircular canal is considered a safe and effective technique for the management of superior semicircular canal dehiscence (SSCD). The aim of this meta-analysis is to assess the postoperative hearing outcomes after the transmastoid plugging of the superior semicircular canal. Search method and data sources: A systematic database search was performed on the following databases until 30 January 2023: MEDLINE, Embase, Cochrane Library, Web of Science, CINAHL, ICTRP, and clinicaltrials.gov. A systematic literature review and meta-analysis of the pooled data were conducted. We also included a consecutive case series with SCDS for those who underwent transmastoid plugging treatment at our clinic. Results: We identified 643 citations and examined 358 full abstracts and 88 full manuscripts. A total of 16 studies were eligible for the systematic review and 11 studies for the meta-analysis. Furthermore, 159 ears (152 patients) were included. The postoperative mean air conduction threshold remained unchanged (mean difference, 2.89 dB; 95% CI: −0.05, 5.84 dB, *p* = 0.58), while the mean bone conduction threshold was significantly worse (mean difference, −3.53 dB; 95% CI, −6.1, −0.95 dB, *p* = 0.9). Conclusion: The transmastoid plugging technique for superior semicircular canal dehiscence syndrome, although minimally worsening the inner ear threshold, is a safe procedure in terms of hearing preservation and satisfactory symptom relief.

## 1. Introduction

Superior semicircular canal dehiscence (SSCD) was initially described in 1998 by Lloyd Minor [1,2,3]. Various techniques have been described for the treatment of this condition [4]. However, vestibular surgery always bears the risk of peri- or postoperative hearing loss due to the loss of perilymph fluid or inflammation. In addition, the traditional surgical access (middle fossa approach) requires a craniotomy and involves some retraction of the temporal lobe [5,6]. Surgery is recommended in patients experiencing disabling vestibular symptoms typically triggered by sound (Tulio phenomenon) and pressure stimuli (Hennebert sign), known as the superior canal dehiscence syndrome (SCDS).

Patients with SCDS often suffer from hearing symptoms such as conductive hyperacusis, phonophobia, autophony, audible eye movements, and pulsatile tinnitus (perceiving rhythmic noises in the ears resembling a heartbeat) [7,8,9].

The surgical repair of the dehiscence involves either plugging, resurfacing, or capping the canal using the middle cranial fossa approach [10,11]. The transmastoid approach, which is a less invasive technique than the middle cranial fossa approach, was first reported by Agrawal and Parnes in 2008 [12]. This approach can be used with both canal plugging and modified resurfacing techniques. The transmastoid approach was modified via assisted endoscopic surgery or underwater plugging techniques [13,14,15]. A balanced salt solution (BSS) might be filled up in the mastoid cavity before a canal repair. BSS has an osmolality similar to periphymph and might prevent the accidental loss of perilymph fluid due to surgical trauma of the membranous labyrinth or due to unintentional suction. The transmastoid access does not provide a direct visualization of the dehiscence over the semicircular canal [12]. Plugging the canal at the ampulated and the non-ampulated end allows for an efficient occlusion of the canal, avoiding any uncontrolled exposure of the dehiscence and manipulations on the middle fossa dura.

The assessment of a surgical complication rate is difficult considering the low prevalence of SCDS in the population (0.4 to 0.5%) and the high rate of patients being asymptomatic.

Although the transmastoid superior semicircular canal plugging technique has demonstrated promising results, it is crucial to conduct a systematic evaluation of its safeness regarding postoperative hearing outcomes and hearing preservation. Therefore, the primary objective of this study was to perform a systematic review of the literature and a meta-analysis to evaluate the impact of transmastoid superior semicircular canal plugging on hearing outcomes and symptom relief in patients diagnosed with SSCD.

## 2. Methods

An initial search strategy was designed by a medical information specialist and complex search strategies were set up for each of the following medical bibliographic databases: MEDLINE, Embase and the Cochrane Library, Web of Science and CINAHL, ICTRP, and clinicaltrials.gov. All searches were run on 30 January 2023 using terms representing: “semicircular canal dehiscence” and “transmastoid plugging” as the intervention.

We restricted our search to publications in English, French, German, Greek, or Italian language. No further limits have been applied to any database concerning study types, languages, or any other formal criteria. The review was conducted following PRISMA Guidelines [16]. We included all retro- and prospective studies (cohort studies or case reports) reporting hearing threshold levels in symptomatic patients with superior semicircular canal dehiscence who underwent transmastoid plugging treatment.

The exact search methodology is reported in Supplementary Material: Section S1. The predefined inclusion and exclusion criteria (coding schema) are reported in the section for the abstract review (Table S1-1) and full-text manuscript review (Table S1-2)

All gathered literature was subject to title/abstract screening by two independent reviewers (ME/RP). Two independent reviewers (ME/RP) applied a full-text screening on abstracts considered eligible or possibly eligible. They assessed and coded all citations for eligibility (Supplementary Material: Section S1, Tables S1-1 and S2-2). Differences were resolved via discussion and consensus. A third reviewer (MG) verified the eligibility of selected articles. Inter-rater agreement on full-text inclusion was calculated using Cohen’s kappa. Risk of bias or applicability concerns were assessed using QUADAS-2 tailored study criteria [17] from two independent raters (ME/RP) (Supplementary Material: Section S2, Table S2-2).

Data extraction for this study was conducted by two authors (EM and PR). The information abstracted from each article encompassed the study type, patient demographics, the number of ears that underwent the procedure, duration of postoperative follow-up, pre- and postoperative pure-tone audiometry thresholds, preoperative symptoms, and the resolution of symptoms postoperatively. All above data are reported in Table 1, Table 2 and Table 3. We contacted the first or corresponding author for additional study information where needed.

For statistical analysis, we used R environment (version 4.2.2, R Core Team) and the meta package (metacont). We used a fixed effect model of meta-analysis for air and bone conduction hearing levels since the studies were considered homogeneous (I^2^ < 50%). A random effect model was used for the analysis of the pre- and postoperative air–bone gap due to the large heterogeneity [18].

**Table 1:** Studies included in the meta-analysis: Study type, patients’ characteristics, and hearing outcomes pre- and postoperative. Mean age is calculated in years, time of postoperative audiometry in months, frequency of thresholds in Hz, pre- and postoperative air and bone conduction thresholds in dB.

Author, Year	Type of Study	Ears	Patients	Mean Age (SD)	Male/ Female	Mean Follow-Up Time	Frequency (kHz)	Mean AC pre-OP (SD)	Mean AC post-OP (SD)	Mean BC pre-OP (SD)	Mean BC post-OP (SD)	Mean ABG pre-OP (SD)	Mean ABG post-OP (SD)
Ellsperman S.E. et al., 2021 [19]	Retrospective	26	26	55 (10.2)	8/18	20	0.5, 1, 2, 4	28.1 (19.6)	26.9 (13.3)	17.9 (16.5)	17.4 (12.9)	22.8 (11.1)	18.7 (9.6)
Kawamura Y. et al., 2022 [15]	Retrospective	7	7	53.3 (13.7)	2/5	12.3	0.5, 1, 2, 4	20.5 (10.6)	20.4 (7.7)	15 (9.1)	18.8 (9.9)	8 (6.4)	3 (5.3)
Gersdorff G. et al., 2022 [20]	Retrospective	30 *	27	52 (10.6)	11/16	19	0.25, 0.5, 1, 2, 3, 4	31.85 (33.3)	29.2 (37.1)	14.5 (23.88)	21.5 (25.5)	15.6 (15.5)	10 (11.8)
Lin K.F. et al., 2021 [21]	Retrospective	29	29	51.2 (NA)	NA	16.4	0.5, 1, 2, 3	25.6 (18.9)	29.5 (20)	17.5 (16)	22.3 (18.4)	8.1 (6.4)	7.2 (7)
Nieto P. et al., 2021 [22]	Retrospective	9	9	52.7 (10)	3/6	9	0.5, 1, 2, 4	23.6 (15.7)	26.4 (14)	15 (13.4)	16.9 (12.6)	NA	NA
Stultiens J.J.A. et al., 2022 [23]	Prospective	4	4	51.5 (6.4)	3/1	2	1, 2, 4	35 (21.8)	38 (20.3)	31.75 (19.6)	33.25 (20.5)	3.25 (2.8)	4.75 (1.9)
Somers T. et al., 2014 [24]	Retrospective	11 *	10	51.5 (14.1)	5/6	7.5	0.5, 1, 2, 4	31.6 (26.7)	40.3 (25.5)	14.8 (19.1)	19.6 (20.6)	23.4 (14.9)	20 (14.9)
Van Haesendonck G. et al., 2016 [25]	Retrospective	13 *	12	51.5 (5.7)	6/7	5.6	0.5, 1, 2, 4	24.3 (12.8)	19.8 (9.5)	13 (9.4)	15.1 (7.1)	12.9 (9.1)	5 (4.7)
Deschenes G.R. et al., 2009 [26]	Retrospective	3 *	2	44.5 (5.5)	2/1	3.5	0.5, 1, 2, 4	18.3 (1.1)	10 (4.6)	10 (0.6)	10.4 (5.2)	9.1 (0.6)	13.85 (8.83)
Morrison M. et al., 2022 + 2 Insel cases [27]	Retrospektive	3	3	53	2/1	2.8	0.5, 1, 2, 4	42 (7.6)	39.1 (2.1)	20 (3)	27.4 (4)	15.8 (10.9)	12.5 (6.7)
Shaul C. et al., 2023 [28]	Retrospective	24 *	23	54	8/15	12	0.5, 1, 2	26.4 (16)	26.5 (19)	13.7 (17)	20.5 (18)	12.7 (8)	5.9 (6)
Cocca S. et al., 2022 ** [29]	Case report	1	1	34	0/1	2	0.5, 1, 2, 4	50	27.5	23.75	22.5	21.3	5
Dang, P.T. et al., 2014 ** [30]	Case report	1	1	52	1/0	9	0.5, 1, 2, 4	41.25	27.5	33.75	23.75	7.5	3.75
Kirtane M.V. et al., 2009 ** [31]	Case report	1	1	37	1/0	2	0.5, 1, 2, 4	31.25	38.75	6.25	22.5	12.5	11.3
Wijaya C. et al., 2012 ** [32]	Case report	1	1	48	1/0	6	0.5, 1, 2, 4	63.75	73.75	45	67.5	17.5	7.5
McCall A.A. et al., 2011 ** [33]	Case report	1	1	15	0/1	12	0.5, 1, 2, 4	5	2.5	−1.25	0	6.3	2.5
Total ***	11 studies	159	152	52.6 (10.2)	50/76	14.1		25.1 (11)	22.2 (10.5)	15.3 (11.2)	18.8 (12.1)	11.5 (10.2)	8.4 (8.6)

* Number of operated ears does not pass the number of patients, as bilateral cases were included in some studies. ** Case reports were not included in the meta-analysis. *** Only studies included in the meta-analysis. SD: standard deviation, audio: audiometry, pre-OP: preoperative, post-OP: postoperative.

**Table 2:** Auditory symptoms before and after surgery.

	Preoperative				Postoperative			
Study	Aural Fullness	Autophony	Tinnitus	Hyperacusis	Aural Fullness	Autophony	Tinnitus	Hyperacusis
Ellsperman S.E. et al., 2021 [19]	NA	NA	NA	NA	NA	NA	NA	NA
Kawamura Y. et al., 2022 [15]	4	6	2	5	0	0	0	NA
Gersdorff G. et al., 2022 [20]	19	29	18	9	1	1	7	0
Lin K.F. et al., 2021 [21]	NA	NA	NA	NA	NA	NA	NA	NA
Nieto P. et al., 2021 [22]	5	7	6	NA	1	0	1	NA
Stultiens J.J.A. et al., 2022 [23]	NA	4	4	NA	NA	0	NA	NA
Somers T. et al., 2014 [24]	NA	9	2	2	NA	0	2	0
Van Haesendonck G. et al., 2016 [25]	NA	1	9	8	NA	1	3	2
Deschenes G.R. et al., 2009 [26]	NA	1	1	NA	NA	NA	NA	NA
Morrison M. et al., 2022 + 2 Insel cases [27]	1	2	2	2	0	0	2	0
Shaul C. et al., 2023 [28]	9	18	16	4	1	1	3	1
Cocca S. et al., 2022 [29]	1	1	NA	1	1	0	1	1
Dang, P.T. et al., 2014 [30]	NA	1	NA	NA	NA	0	NA	NA
Kirtane M.V. et al., 2009 [31]	1	NA	1	NA	0	NA	NA	NA
Wijaya C. et al., 2012 [32]	0	NA	0	NA	0	NA	0	NA
Total (rate of patients with remaining symptoms)	41	79	62	31	4 (9.7%)	3 (3.7%)	19 (30%)	4 (12.9%)

NA: not available.

**Table 3:** Vestibular symptoms before and after surgery.

	Preoperative			Postoperative		
Study	Tullio Sign	Hennebert Sign	Oscillopsia	Tullio Sign	Hennebert Sign	Oscillopsia
Ellsperman S.E. et al., 2021 [19]	NA	NA	NA	NA	NA	NA
Kawamura Y. et al., 2022 [15]	2	1	NA	NA	0	NA
Gersdorff G. et al., 2022 [20]	19	NA	11	2	NA	2
Lin K.F. et al., 2021 [21]	NA	NA	NA	NA	NA	NA
Nieto P. et al., 2021 [22]	0	4	6	NA	0	1
Stultiens J.J.A. et al., 2022 [23]	NA	NA	NA	NA	NA	NA
Somers T. et al., 2014 [24]	3	NA	1	0	NA	0
Van Haesendonck G. et al., 2016 [25]	5	NA	NA	1	NA	NA
Deschenes G.R. et al., 2009 [26]	1	1	NA	0	0	NA
Morrison M. et al., 2022 + 2 Insel cases [27]	2	1	1	0	0	0
Shaul C. et al., 2023 [28]	15	NA	4	0	NA	0
Cocca S. et al., 2022 [29]	0	1	NA	NA	0	NA
Dang, P.T. et al., 2014 [30]	1	1	1	0	0	0
Kirtane M.V. et al., 2009 [31]	NA	1	NA	NA	0	NA
Wijaya C. et al., 2012 [32]	0	1	NA	0	0	NA
Total (rate of patients with remaining symptoms)	48	11	24	3 (6%)	0 (0%)	3 (12.5%)

NA: not available.

## 3. Results

### 3.1. Search Results and Study Selection

**Figure 1:**
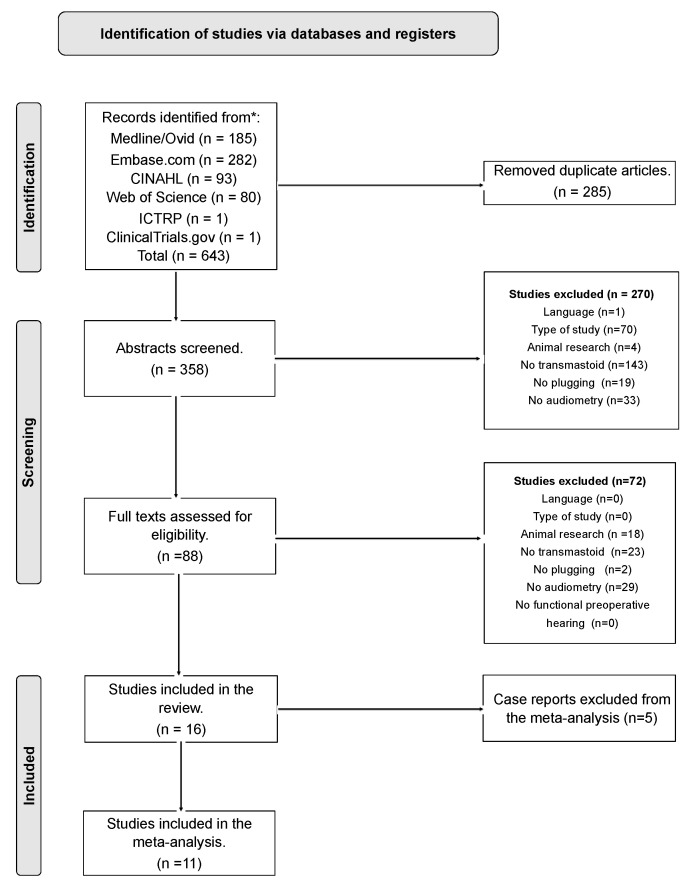
Prisma flow chart.

The PRISMA flow chart in Figure 1 illustrates the search and review process. Our search identified 643 unique citations, and we sought to examine 358 full abstracts, of which 270 were excluded at the abstract level. After initial screening, there were a total of 23 disagreements about study inclusion from the two reviewers (ME and RP, inter-rater reliability κ = 0.814). Overall disagreement on the reason for exclusion was 18%. These differences were resolved via discussion and adjudication by a third reviewer (MG).

We performed a full-text screening on 88 manuscripts (disagreement 1.4%, inter-rater reliability κ  = 0.885). We included 16 eligible articles (9 retrospective studies, 1 prospective study, and 6 case reports, as shown in Table 1), representing 2.3% of the total (*n* = 643).

We included 11 out of 16 publications in the meta-analysis. Five case reports were excluded from the meta-analysis due to selection bias. We included our own case report from Morrison et al. [27] which was part of an unbiased consecutive case series (*n* = 3) conducted from August 2020 to September 2022. For included studies, two independent raters (ME/RP) assessed the risk of bias or applicability concerns using QUADAS-2 tailored study criteria [17], resolving disagreements via discussion. Two studies were rated as having a high risk of bias/applicability concerns in the domain of “flow and timing”, as the postoperative audiogram was performed more than 18 months after surgery.

The I^2^ statistic, indicating the degree of heterogeneity among the included studies, was 2% for AC and 0% for BC. On the other hand, the I^2^ statistic for the air–bone gap was 50%, representing substantial heterogeneity in the studies [34].

### 3.2. Characteristics of Patients

Overall, 159 ears (152 patients) received a transmastoid canal plugging and were included in the study. Six patients underwent bilateral surgery, and one patient was operated on twice. The calculated mean age was 52.6 years (SD 10.2). The male/female ratio was around 0.7 (Table 1). In one study the exact male/female ratio was not available.

The mean follow-up time was 52.6 years. The average monitoring period was approximately 14 months. Out of the total, 56 patients were followed for a duration exceeding 18 months, whereas only seven ears were reported to have been within a brief time frame of just 2 months.

### 3.3. Hearing Outcomes

No cases of total hearing loss were reported after transmastoid canal plugging. However, one patient in the study of Ellsperman et al. [19] exhibited a significant sensorineural hearing loss of 30 dB eight months after the surgery.

**Figure 2:**
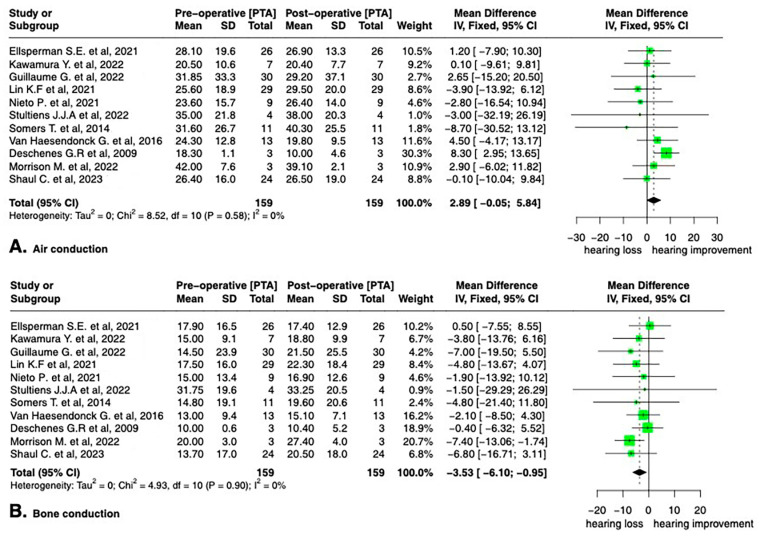
Forest plot for the comparison of mean threshold for air conduction (**A**) and bone conduction (**B**) after transmastoid canal plugging on patients with semicircular canal dehiscence syndrome [15,19,20,21,22,23,24,25,26,27,28].

We found a mean air conduction (AC) threshold at 25.1 dB PTA (SD 11) preoperatively and 22.2 dB (SD 10.5) postoperatively (Table 2). AC hearing thresholds did not significantly change after surgery (2.89 dB; 95% CI −0.05, 5.84 dB; I^2^ = 0%) (Figure 2A).

We found a mean bone conduction threshold at 15.3 dB PTA (SD 11.2) preoperatively and 18.8 dB (SD 12.1) postoperatively (Table 2). The mean preoperative BC threshold decreased significantly by −3.53 dB (95% CI, −6.10, −0.95 dB; I^2^ = 0%) from 15.8 dB (SD 17.1) to 19.9 dB (SD 17.7) (Figure 2B).

**Figure 3:**
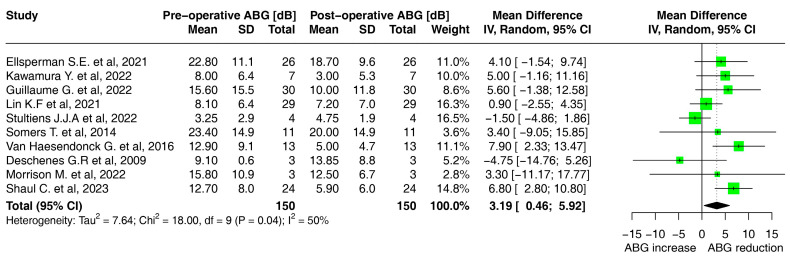
Forest plot for the comparison of mean air–bone gap after transmastoid canal plugging on patients with semicircular canal dehiscence syndrome [15,19,20,21,22,23,25,26,27,28].

The air–bone gap before and after surgery was also studied in a subanalysis (Table 2); in one study, the air–bone gap data were not available. The mean preoperative air–bone gap was calculated to be 11.5 dB (SD 10.2) and decreased by 3.19 dB (SD 2.7) to 8.4 dB (8.6 SD) (Figure 3).

### 3.4. Clinical Symptoms before and after Surgery

Table 2 and Table 3 summarize information about the auditory and vestibular symptoms of the patients both before and after surgery. Data from the case reports were also included in these tables. Unfortunately, not all articles reported preoperative clinical symptoms and postoperative rates of symptom resolution. Autophony and tinnitus were the most reported hearing symptoms among the patients; aural fullness and hyperacusis were less frequently complained about. Following surgery, there was a significant improvement in all auditory symptoms, with an improvement rate exceeding 90% for most symptoms, except for tinnitus, which persisted in 30% of the cases. (Table 2).

Regarding vestibular symptoms and signs, the patients predominantly reported oscillopsia, and the Tullio phenomenon was frequently documented. Less frequently documented was a positive Hennebert’s sign. Postoperatively, there was a high recovery rate of symptoms, with a resolution of the Tulio sign in 90.3%, Hennebert sign in 100%, and oscillopsia in 87.5% (Table 3).

### 3.5. Surgical Complications

The most common complication postoperative was nausea, vertigo, or unsteadiness, which resolved over time. One patient suffered from a benign paroxysmal positional vertigo attack, which was successfully treated with the repositioning maneuver.

We searched the included studies for possible surgical complications such as hemorrhage, epidural damage or bleeding, neurological complications, pneumolabyrinth, complete labyrinth failure, and infections, but no such complication was reported in any case.

## 4. Consecutive Case Series

We present here three SCDS cases from an unbiased consecutive case series of patients who underwent transmastoid semicircular plugging at our clinic from August 2020 to September 2022. One of these patients, a 66-year-old man, was referred to our clinic for hyperacusis, tinnitus, autophonia, and balance disorder, including oscillopsia. He had conductive hearing loss, which was unsuccessfully addressed surgically via oval window re-enforcement many decades ago. He had a rare sign of fremitus nystagmus (a mixed downbeat and torsional nystagmus elicited by sustained humming), which was recorded and published by our group [27]. His pre-existing tinnitus and conductive hearing loss neither improved nor worsened after surgery.

The second patient was a 37-year-old man presenting with subjective hearing loss, tinnitus, sound-induced vertigo, and positive Hennebert sign. He lost his postural stability upon the Valsalva maneuver and had a falling tendency in the direction of the left superior canal plane. The third patient, a 56-year-old woman, presented with autophony, hyperacusis, pulsatile tinnitus, and vertigo. Both patients complained about audible body sounds, exhibited negative Tullio, and were free of nystagmus. Following the surgical procedure, both patients had a complete resolution of all clinical symptoms, except tinnitus.

Table 1 and Figure 2 and Figure 3 show the pre- and post-operative hearing results of the three patients.

## 5. Discussion

Our meta-analysis shows that the transmastoid plugging technique for superior semicircular canal dehiscence (SSCD) syndrome appears to be linked to positive preservation of air conduction thresholds and a significant reduction in the air–bone gap after the surgery. Despite the postoperative bone conduction showing statistically worse results when compared to the preoperative values, the thresholds still remained within the normal hearing range [13].

### 5.1. Hearing Preservation after Plugging

Since the plugging technique requires a double opening of the semicircular canal at the ampullary and non-ampullary end and a more forceful manipulation of the membranous canal, the risk of permanent or transient sensorineural hearing loss, important disequilibrium or benign paroxysmal vertigo, or even membranous canal rupture is much higher [35]. In our meta-analysis, though, there were no such complications reported. Only one patient suffered from a benign paroxysmal positional vertigo attack, which was successfully treated with the repositioning maneuver.

Additionally, according to Gioacchini et al., there was no statistically significant correlation between the complications mentioned above and the used technique: plugging, resurfacing, and caping [36].

Furthermore, via the transmastoid approach, there is a potential risk of perilymph aspiration when the direct visualization of the dehiscence is not possible. However, this can be counteracted with the underwater endoscopic ear surgery technique. This technique provides a clear operative field without requiring suction and protects the inner ear from unexpected aeration that may damage its function [13].

### 5.2. Symptoms Resolution

In our study, we observed a subjective improvement in patients after surgery, primarily concerning auditory symptoms like aural fullness, autophony, hyperacusis, and vestibular symptoms, including vertigo and oscillopsia. We also observed a decrease in Tullio and Hennebert signs after surgery. However, the symptom that showed the lowest rate of improvement was tinnitus. The rates of symptom resolution are shown in Table 2 and Table 3. These findings align with the study conducted by Allsopp et al., which demonstrated a notable decrease in the overall Dizziness Handicap Index, as well as an effective improvement in autophony, dizziness, and overall quality of life following the transmastoid repair of superior semicircular canal dehiscence [37].

### 5.3. Surgical Approach and Complications

There is a substantial body of literature regarding the safety and effectiveness of the middle fossa approach, while the transmastoidal approach offers numerous benefits. The middle cranial fossa approach has advantages when the dehiscence may not be visualized due to the relatively low positioning of the middle fossa dura. Additionally, extensive cranial base dehiscences that coexist may also necessitate reconstruction, which is most effectively, accomplished using the middle fossa approach [12,38]. The transmastoid approach, however, does not require craniotomy or temporal lobe retraction and is, therefore, less invasive than the middle fossa approach. A systematic review conducted by Ziylan et al. reached the conclusion that transmastoid dehiscence plugging was associated with reduced hospital stay, complication rates, and revision rates compared to the middle cranial fossa MCF approach [4]. The above aligns with the complicated outcomes presented in our meta-analysis. Additionally, the occlusion of the canal can be achieved without initially manipulating the dehiscence [12].

### 5.4. Limitations

To our knowledge, this review is the first to report pooled hearing outcomes after plugging of semicircular canal dehiscence via transmastoid approach and assess the efficacy of the technique in relation to postoperative hearing outcomes and hearing preservation, guiding future research and aiding clinicians in making informed treatment decisions.

A significant limitation is the limited number of patients included in this review. This constraint arises due to the infrequent prevalence of superior semicircular canal dehiscence (SSCD) syndrome.

We observed a lack of consensus regarding the recommended duration of follow-up needed to assess the potential success of surgery. The follow-up time and time of the post-operative hearing test were heterogeneous.

It is important to note all included studies except one were retrospective in nature.

### 5.5. Future Implications

The future implications of the transmastoid plugging technique hold significant promise in the field of otology and neurotology. The transmastoid οpening of the semicircular canals without plugging will be relevant in the future for electrode insertion in vestibular implant systems, ultimately enhancing their precision and effectiveness. In patients with normal hearing and bilateral vestibulopathy, the preservation of hearing should be prioritized to the greatest extent possible.

Moreover, as our understanding of the underlying pathophysiology of semicircular dehiscence deepens, tailored approaches to patient selection and individualized treatment strategies may emerge, optimizing outcomes and minimizing complications. Collaboration between clinicians, researchers, and industry experts can further drive the development of novel materials and implants, fostering continual progress in the field.

## 6. Conclusions

The transmastoid plugging technique for superior semicircular canal dehiscence syndrome is a safe procedure in terms of hearing preservation and satisfactory symptom relief. Due to the limited sample size, we recommend further prospective multicenter studies in the future that systematically assess hearing and dizziness symptoms.

## Data Availability

No new data were created.

## Supplementary Materials

The following supporting information can be downloaded at: https://www.mdpi.com/article/10.3390/audiolres13050065/s1. Section S1: electronic search strategy, coding-scheme for the systematic review and data analysis, Table S1-1: Abstract reasons for exclusion, Table S1-2: Full-Text reasons for exclusion, Section S2: QUADAS-2 assessment of included studies, Table S2-1: QUADAS-2 quality ratings for included studies (*n* = 11).
