# Supplementary material for: Hearing Results after Transmastoid Superior Semicircular Canal Plugging for Superior Semicircular Canal Dehiscence: A Meta-Analysis
Source: Audiol Res. 2023 Oct 8;13(5):730–40. doi: 10.3390/audiolres13050065 (PMC10604912; doi:10.3390/audiolres13050065)
Supplement: Supplementary file 1 [file audiolres-13-00065-s001.zip › audiolres-2637322-supplementary.pdf]

Supplementary material

Table of contents

Section S1. electronic search strategy, coding-scheme for the systematic review and data analysis
Table S1-1: Abstract reasons for exclusion
Table S1-2: Full-Text reasons for exclusion

Section S2. QUADAS-2 assessment of included studies
Table S2-1: QUADAS-2 quality ratings for included studies (n=11)

References

Section S1: Electronic search strategy, coding-scheme for the systematic review and data analysis

An initial search strategy was developed in Medline/Ovid by a medical information specialist and tested against a list of core references to see if they were included in the search result. After refinement and consultation, complex search strategies were set up for each information source based on database-specific controlled vocabulary (thesaurus terms / subject headings) and textwords. Synonyms, acronyms, and similar terms were included in the textword search. The medical bibliographic databases MEDLINE, Embase and the Cochrane Library, Web of Science and CINAHL as well as the clinical trial registries ICTRP and clinicaltrials.gov were searched. All searches were run on 30th of January 2023.

Limits have been applied to publication of English, French, German, Greek or Italien language. No further limits have been applied in any database considering study types, languages, or any other formal criteria.

The following search concepts were applied according to the PICO framework: 1. "semicircular canal dehiscence" as the population/problem, 2. "transmastoid plugging" as the intervention, 3. no comparison was applied, 4. no outcome was applied. Synonyms, acronyms, and similar terms were used for all concepts in the textword search, as well as the respective thesaurus terms.

Ovid MEDLINE(R) ALL <1946 to January 30, 2023>
Search Date: 31/01/2023

1 exp Semicircular Canal Dehiscence/ 75
2 (((Semicircular or semi-circular) adj4 canal* adj4 dehiscence*) or (canal* adj4 dehiscence* adj4 syndrome*) or tullio phenomen* or sscd or scds).mp. 1368
3 1 or 2 1368
4 exp Semicircular Canals/su 466
5 ((transmastoid* or tm) and (management or approach* or repair* or plug* or surfac* or resurf* or cap* or surg* or technique? or occlus* or occlud* or blog* or clog*)).mp. 34856
6 4 or 5 35256
7 3 and 6191
8 limit 7 to (english or french or german or greek or italian) 185

Embase.com (1947 to 30 January 2023)
Search Date: 31/01/2023

#9. #7 AND #8 282

#8. [english]/lim OR [french]/lim OR [german]/lim OR [greek]/lim OR [italian]/lim 38,834,416

#7. #3 AND #6 286

#6. #4 OR #5 25,241

#5. (transmastoid*:ti,ab,kw,de OR tm:ti,ab,kw,de) AND (management:ti,ab,kw,de OR approach*:ti,ab,kw,de OR repair*:ti,ab,kw,de OR plug*:ti,ab,kw,de OR surfac*:ti,ab,kw,de OR resurf*:ti,ab,kw,de OR cap*:ti,ab,kw,de OR surg*:ti,ab,kw,de OR technique*:ti,ab,kw,de OR occlus*:ti,ab,kw,de OR occlud*:ti,ab,kw,de OR blog*:ti,ab,kw,de OR clog*:ti,ab,kw,de) 24,033

#4. 'semicircular canal'/exp AND 'surgery'/exp 1,321

#3. #1 OR #2 2,135

#2. (((semicircular OR 'semi-circular') NEAR/4 canal* NEAR/4 dehiscence*):ab,kw,ti,de) OR ((canal* NEAR/4 dehiscence* NEAR/4 syndrome*):ab,kw,ti,de) OR 'tullio phenomen*':ab,kw,ti,de OR sscd:ab,kw,ti,de OR scds:ab,kw,ti,de 2,135

#1. 'semicircular canal dehiscence'/exp 173

Cochrane Library <1996 to present>
Search Date: 31/01/2023

#1 [mh "Semicircular Canal Dehiscence"] 1

#2 ((Semicircular:ti,ab,kw OR semi-circular:ti,ab,kw) NEAR/4 (canal*:ti,ab,kw NEAR/4 dehiscence*:ti,ab,kw)) OR (canal*:ti,ab,kw NEAR/4 dehiscence*:ti,ab,kw NEAR/4 syndrome*:ti,ab,kw) OR ("tullio" NEXT phenomen*):ti,ab,kw OR sscd:ti,ab,kw OR scds:ti,ab,kw 45

#3 #1 OR #2 45

#4 [mh "Semicircular Canals"/su] 4

#5 (transmastoid* or tm):ti,ab,kw AND (management OR approach* OR repair* OR plug* OR surfac* OR resurf* OR cap* OR surg* OR technique*):ti,ab,kw 2778

#6 #4 OR #5 2782

#7 #3 AND #6 0

Web of Science Core Collection (Clarivate Analytics) (1900-2023)
(Science Citation Index Expanded: WOS.SCI: 1900 to 2023, WOS.AHCI: 1975 to 2023, WOS.ESCI: 2018 to 2023, WOS.ISTP: 1990 to 2023, WOS.SSCI: 1900 to 2023, WOS.ISSHP: 1990 to 2023)
Search Date: 31/01/2023

1	TS=((transmastoid* OR tm) AND (management OR approach* OR repair* OR plug* OR surfac* OR resurf* OR cap* OR surg* OR technique$ OR occlus* OR occlud* OR blog* OR clog*))	81961
2	TS=((Semicircular OR semi-circular) NEAR/4 canal* NEAR/4 dehiscence*)	666
3	TS=(canal* NEAR/4 dehiscence* NEAR/4 syndrome*)	272

4	TS=("tullio phenomen*" OR sscd OR scds)	1332
5	#2 OR #3 OR #4	1797
6	#1 AND #5	80

CINAHL (via EBSCO) (1982 to 30 January 2023)
Search Date: 31/01/2023

S8	S3 AND S6	93	Narrow by Language: - english Search modes - Find all my search terms
S7	S3 AND S6	93	Search modes - Find all my search terms
S6	S4 OR S5	1,820	Search modes - Find all my search terms
S5	TI (((transmastoid* OR tm) AND (management OR approach* OR repair* OR plug* OR surfac* OR resurf* OR cap* OR surg* OR technique# OR occlus* OR occlud* OR blog* OR clog*))) OR AB (((transmastoid* OR tm) AND (management OR approach* OR repair* OR plug* OR surfac* OR resurf* OR cap* OR surg* OR technique# OR occlus* OR occlud* OR blog* OR clog*)))	1,706	Search modes - Find all my search terms
S4	(MH "Semicircular Canals/SU")	141	Search modes - Find all my search terms
S3	S1 OR S2	4,524	Search modes - Find all my search terms
S2	TI ((((Semicircular OR semi-circular) N4 canal* N4 dehiscence*) OR (canal* N4 dehiscence* N4 syndrome*) OR "tullio phenomen*" OR sscd OR scds)) OR AB ((((Semicircular OR semi-circular) N4 canal* N4 dehiscence*) OR (canal* N4 dehiscence* N4 syndrome*) OR "tullio phenomen*" OR sscd OR scds))	4,509	Search modes - Find all my search terms
S1	(MH "Semicircular Canal Dehiscence") OR (MH "Superior Semicircular Canal Dehiscence Syndrome")	77	Search modes - Find all my search terms

The World Health Organization (WHO) International Clinical Trials Registry Platform (ICTRP) Search Date: 31/01/2023

Advanced Searches:

1.	Title:	canal dehiscence*	Recruitment status: ALL
2.	Condition:	canal dehiscence*	Recruitment status: ALL
3.	1. OR 2. 1		

Clinicaltrials.gov
Search Date: 31/01/2023

1. Condition or disease: canal dehiscence* 1

Recruitment status: All studies
Recruitment: All studies
Study results: All studies
Study type: All studies

Inclusion and exclusion rules for abstracts & full-text manuscripts

Our search identified 643 unique citations and we sought to examine 358 full abstracts, of which 270 were excluded at the abstract level (see PRISMA flow chart). After initial screening, there were a total of 23 disagreements about study inclusion for the two reviewers (ME and RP, kappa= 0.814). These differences were resolved by discussion and adjudication by a third reviewer. Overall disagreement on reason for exclusion was 18%. We demanded concordance on reason for full-text exclusion and resolved differences by discussion.

Among the excluded abstracts, the distribution was as follows: 1 excluded due to language, 70 excluded due to type of study, 4 were animal research, 143 were not about transmastoid approach of superior semicircular canal; 19 were not about plugging the superior semicircular canal; 33 did not include audiometry results.

We sought to examine 88 full manuscripts. After initial screening, there were a total of 5 disagreements about study inclusion for the two reviewers (ME and RP, kappa=0.885). These differences were resolved by discussion and adjudication by a third reviewer. Overall disagreement on reason for exclusion was 1.4%. We demanded concordance on reason for full-text exclusion and resolved differences by discussion.

At the end of our full-text review, 72 were excluded and 16 were considered eligible (see PRISMA flow chart Figure 1). These eligible studies represented 2.3% of the total (n=643). Among all full-text manuscripts excluded, the distribution of reason for exclusion was as follows: 18 excluded due to type of study, 23 were not about transmastoid approach of superior semicircular canal; 2 were not about plugging the superior semicircular canal; 29 did not include pre-and postoperative pure-tone audiometry thresholds.

For 24 studies we attempted to contact the first or corresponding author for additional study information. 6 authors responded and 2 provided additional information.

Abstract review coding rules

1) Coding status options are “Yes”, “No”, “Maybe”. We will review full text of “Yes” and “Maybe”.
2) Err on the side of “Maybe” if there is doubt about a “No”; this is more conservative.
3) If there is only a title, exclude it only if you feel confident; otherwise, code it as “Maybe”.
4) Each "No" should be coded with a reason for exclusion.
5) Reasons for exclusion are listed below 0-5. Go through them in order from 0 to 5 for each abstract, coding the first reason for exclusion only, not multiple reasons for exclusion.
6) Two independent raters (ME/RP) will code reason for exclusion, and we will mandate agreement on exclusion reason at the abstract level.
7) Coding differences will be adjudicated, or consensus will be developed through dialogue.

1	Language	Other Language than English, Greek, Italian, French or German
2	Type of study	Study other than retrospective and prospective cohort studies or case reports
3	Animal research	Animal-surgery
4	No transmastoid	No reasonable prospect that the study includes transmastoid surgical access of the superior semicircular canal
5	No plugging	No reasonable prospect that the study includes semicircular plugging
6	No audiometry	No reasonable prospect that the study includes pre-and postoperative pure-tone audiometry thresholds

Table S1-1: Abstract reasons for exclusion.

Full-text review coding rules
1) Coding status options are "Yes" or "No".
2) Each "No" should be coded with a reason for exclusion.
3) Reasons for exclusion are listed below 0-5. Go through them in order from 0 to 5 for each full manuscript, coding the first reason for exclusion only, not multiple reasons for exclusion.
4) Two independent raters (ME/RP) will code reason for exclusion, and we will mandate agreement on exclusion reason at the manuscript level.
5) Coding differences will be adjudicated, or consensus will be developed through dialogue.

1	Language	Other Language than English, Greek, Italian, French or German
2	Type of study	Study other than retrospective and prospective cohort studies or case reports
3	Animal research	Animal-surgery
4	No transmastoid	No transmastoid surgical access of the superior semicircular canal
5	No plugging	No semicircular plugging performed
6	No audiometry	The study doesn't include pre-and postoperative pure-tone audiometry thresholds
7	No functional preoperative hearing	No functional preoperative hearing

Table S1-2: Full-text reasons for exclusion.

Section S2: QUADAS-2 assessment of included studies

For included studies, two independent raters (ME/PR) assessed the risk of bias or applicability concerns using QUADAS-2 tailored study criteria, resolving disagreements by discussion. The study of Morrison et al [16] was in included in the meta-analysis, despite being a case report, as it was combined with a case series that included two additional patients from our clinic.

The QUADAS-2 tool for quality rating of diagnostic accuracy studies consists of four core domains (patient selection, index test, reference standard, and flow and timing). Reference standard was not applicable for our study. Risk of bias is assessed for all three domains (patient selection. Index test and flow and timing), and applicability is assessed for the first two domains. Thus, five items per study are assessed to rate quality of evidence.

Two studies were rated as having high risk of bias/applicability concerns in the domain of "flow and timing", as the postoperative audiogram was performed more than 18 months after surgery (see Table S2-1). However, both trials were included in the meta-analysis.

Author, Year	Sample Size	Patients' selection		Index Test		Flow and Timing
		Risk of bias	Applicability	Risk of bias	Applicability	Risk of bias
Ellsperman S.E. et al, 2021 [19]	26	low risk*	low risk	low risk	low risk	high risk
Kawamura Y. et al, 2022 [15]	7	low risk	low risk	low risk	low risk	low risk
Gersdorff G. et al, 2022 [20]	30	low risk	low risk	low risk	low risk	high risk
Lin K.F et al, 2021 [21]	29	low risk	low risk	low risk	low risk	low risk
Nieto P. Et al, 2021 [22]	9	low risk	low risk	low risk	low risk	low risk
Stultiens J.J.A et al, 2022 [23]	4	low risk	low risk	low risk	low risk	low risk
Somers T. Et al, 2014 [24]	11	low risk	low risk	low risk	low risk	low risk
Van Haesendonck G. et al, 2016 [25]	13	low risk	low risk	low risk	low risk	low risk
Deschenes G.R. et al, 2009 [26]	3	low risk	low risk	low risk	low risk	low risk
Morrison M. et al, 2022+2 Insel cases [27]	3	low risk	low risk	low risk	low risk	low risk
Shaul C. et al, 2023 [28]	24	low risk	low risk	low risk	low risk	low risk

Table S2-1: QUADAS-2 quality ratings for included studies (n=11) .

*2 Patients were exluded from the study of Ellsperman et al because there was no available postoperative audiogram. The study was rated, though, as low risk as this type of exclusion has no systematic impact on the results of the study that deviate from the truth.

References

1. Ward, B.K.; van de Berg, R.; van Rompaey, V.; Bisdorff, A.; Hullar, T.E.; Welgampola, M.S.; Carey, J.P. Superior Semicircular Canal Dehiscence Syndrome: Diagnostic Criteria Consensus Document of the Committee for the Classification of Vestibular Disorders of the Bárány Society. J. Vestib. Res. Equilib. Orientat. 2021, 31, 131–141. https://doi.org/10.3233/VES-200004.
2. Minor, L.B.; Solomon, D.; Zinreich, J.S.; Zee, D.S. Sound- and/or Pressure-Induced Vertigo Due to Bone Dehiscence of the Superior Semicircular Canal. Arch. Otolaryngol.—Head Neck Surg. 1998, 124, 249–258. https://doi.org/10.1001/archotol.124.3.249.
3. Minor, L.B. Clinical Manifestations of Superior Semicircular Canal Dehiscence. Laryngoscope 2005, 115, 1717–1727. https://doi.org/10.1097/01.mlg.0000178324.55729.b7.
4. Ziylan, F.; Kinaci, A.; Beynon, A.J.; Kunst, H.P.M. A Comparison of Surgical Treatments for Superior Semicircular Canal Dehiscence: A Systematic Review. Otol. Neurotol. 2017, 38, 1–10. https://doi.org/10.1097/MAO.0000000000001277.
5. Ward, B.K.; Carey, J.P.; Minor, L.B. Superior Canal Dehiscence Syndrome: Lessons from the First 20 Years. Front. Neurol. 2017, 8, 177. https://doi.org/10.3389/fneur.2017.00177.
6. Steenerson, K.K.; Crane, B.T.; Minor, L.B. Superior Semicircular Canal Dehiscence Syndrome. Semin. Neurol. 2020, 40, 151–159. https://doi.org/10.1055/s-0039-3402738.
7. Schmuziger, N.; Allum, J.; Buitrago-Téllez, C.; Probst, R. Incapacitating Hypersensitivity to One's Own Body Sounds Due to a Dehiscence of Bone Overlying the Superior Semicircular Canal. A Case Report. Eur. Arch. Oto-Rhino-Laryngol. 2006, 263, 69–74. https://doi.org/10.1007/s00405-005-0939-9.
8. Minor, L.B.; Carey, J.P.; Cremer, P.D.; Lustig, L.R.; Streubel, S.-O.; Ruckenstein, M.J. Dehiscence of Bone Overlying the Superior Canal as a Cause of Apparent Conductive Hearing Loss. Otol. Neurotol. 2003, 24, 270–278. https://doi.org/10.1097/00129492-200303000-00023.
9. Watson, S.R.; Halmagyi, G.M.; Colebatch, J.G. Vestibular Hypersensitivity to Sound (Tullio Phenomenon). Neurology 2000, 54, 722–728. https://doi.org/10.1212/WNL.54.3.722.
10. Vlastarakos, P.V.; Proikas, K.; Tavoulari, E.; Kikidis, D.; Maragoudakis, P.; Nikolopoulos, T.P. Efficacy Assessment and Complications of Surgical Management for Superior Semicircular Canal Dehiscence: A Meta-Analysis of Published Interventional Studies. Eur. Arch. Oto-Rhino-Laryngol. 2009, 266, 177–186. https://doi.org/10.1007/s00405-008-0840-4.
11. Mueller, S.A.; Vibert, D.; Haeusler, R.; Raabe, A.; Caversaccio, M. Surgical Capping of Superior Semicircular Canal Dehiscence. Eur. Arch. Oto-Rhino-Laryngol. 2013, 271, 1369–1374. https://doi.org/10.1007/s00405-013-2533-x.
12. Agrawal, S.K.; Parnes, L.S. Transmastoid Superior Semicircular Canal Occlusion. Otol. Neurotol. 2008, 29, 363–367. https://doi.org/10.1097/mao.0b013e3181616c9d.
13. Yamauchi, D.; Yamazaki, M.; Ohta, J.; Kadowaki, S.; Nomura, K.; Hidaka, H.; Oshima, T.; Kawase, T.; Katori, Y. Closure Technique for Labyrinthine Fistula by 'Underwater' Endoscopic Ear Surgery. Laryngoscope 2014, 124, 2616–2618. https://doi.org/10.1002/lary.24785.
14. Creighton, F.X.J.; Zhang, L.; Ward, B.; Carey, J.P. Hearing Outcomes for an Underwater Endoscopic Technique for Transmastoid Repair of Superior Semicircular Canal Dehiscence. Otol. Neurotol. 2021, 42, E1691–E1697. https://doi.org/10.1097/MAO.0000000000003238.
15. Kawamura, Y.; Yamauchi, D.; Kobayashi, T.; Ikeda, R.; Kawase, T.; Katori, Y. Hearing Outcomes of Transmastoid Plugging for Superior Canal Dehiscence Syndrome by Underwater Endoscopic Surgery: With Special Reference to Transient Bone Conduction Increase in Early Postoperative Period. Otol. Neurotol. 2022, 43, 368–375. https://doi.org/10.1097/MAO.0000000000003461.
16. Page, M.J.; McKenzie, J.E.; Bossuyt, P.M.; Boutron, I.; Hoffmann, T.C.; Mulrow, C.D.; Shamseer, L.; Tetzlaff, J.M.; Akl, E.A.; Brennan, S.E.; et al. The PRISMA 2020 Statement: An Updated Guideline for Reporting Systematic Reviews. J. Clin. Epidemiol. 2021, 134, 178–189. https://doi.org/10.1016/j.jclinepi.2021.03.001.
17. Whiting, P.F.; Rutjes, A.W.S.; Westwood, M.E.; Mallett, S.; Deeks, J.J.; Reitsma, J.B.; Leeflang, M.M.G.; Sterne, J.A.C.; Bossuyt, P.M.M.; QUADAS-2 Group. QUADAS-2: A Revised Tool for the Quality Assessment of Diagnostic Accuracy Studies. Ann. Intern. Med. 2011, 155, 529–536.
18. Balduzzi, S.; Rücker, G.; Schwarzer, G. How to Perform a Meta-Analysis with R: A Practical Tutorial. Evid. Based Ment. Health 2019, 22, 153–160. https://doi.org/10.1136/ebmental-2019-300117.
19. Ellsperman, S.E.; Telian, S.A.; Kileny, P.R.; Welch, C.M. Auditory Outcomes Following Transmastoid and Middle Cranial Fossa Approaches for Superior Semicircular Canal Dehiscence Repair. Otol. Neurotol. 2021, 42, 1544–1552. https://doi.org/10.1097/MAO.0000000000003323.
20. Gersdorff, G.; Blaivie, C.; de Foer, B.; Deggouj, N.; Wyckmans, F.; Somers, T. Evaluation of the Transmastoid Plugging Approach for Superior Semicircular Canal Dehiscences: A Retrospective Series of 30 Ears. Eur. Arch. Oto-Rhino-Laryngol. 2022, 279, 4861–4869. https://doi.org/10.1007/s00405-022-07316-8.
21. Lin, K.F.; Bojrab, D.I.I.; Fritz, C.G.; Vandieren, A.; Babu, S.C. Hearing Outcomes After Surgical Manipulation of the Membranous Labyrinth During Superior Semicircular Canal Dehiscence Plugging or Posterior

Semicircular Canal Occlusion. Otol. Neurotol. 2021, 42, 806–814. https://doi.org/10.1097/MAO.0000000000003100.
22. Nieto, P.; Gallois, Y.; Molinier, C.; Deguine, O.; Marx, M. Surgical Treatments of Superior Semicircular Canal Dehiscence: A Single-Centre Experience in 63 Cases. Laryngoscope Investig. Otolaryngol. 2021, 6, 1414–1420. https://doi.org/10.1002/lio2.684.
23. Stultiens, J.J.A.; Guinand, N.; Van Rompaey, V.; Fornos, A.P.; Kunst, H.P.M.; Kingma, H.; van de Berg, R. The Resilience of the Inner Ear—Vestibular and Audiometric Impact of Transmastoid Semicircular Canal Plugging. J. Neurol. 2022, 269, 5229–5238. https://doi.org/10.1007/s00415-021-10693-5.
24. Zhao, Y.C.; Somers, T.; van Dinther, J.; Vanspauwen, R.; Husseman, J.; Briggs, R. Transmastoid Repair of Superior Semicircular Canal Dehiscence. J. Neurol. Surg. Part B Skull Base 2012, 73, 225–229. https://doi.org/10.1055/s-0032-1312713.
25. Van Haesendonck, G.; Van de Heyning, P.; Van Rompaey, V. Retrospective Cohort Study on Hearing Outcome after Transmastoid Plugging in Superior Semicircular Canal Dehiscence Syndrome: Our Experience. Clin. Otolaryngol. 2016, 41, 601–606. https://doi.org/10.1111/coa.12539.
26. Deschenes, G.R.; Hsu, D.P.; Megerian, C.A. Outpatient Repair of Superior Semicircular Canal Dehiscence via the Transmastoid Approach. Laryngoscope 2009, 119, 1765–1769. https://doi.org/10.1002/lary.20543.
27. Morrison, M.; Korda, A.; Wagner, F.; Caversaccio, M.D.; Mantokoudis, G. Case Report: Fremitus Nystagmus in Superior Canal Dehiscence Syndrome. Front. Neurol. 2022, 13, 844687. https://doi.org/10.3389/fneur.2022.844687.
28. Shaul, C.; Weder, S.; Dragovic, A.; Gerard, J.-M.; Briggs, R.J.S. Trans-Mastoid Plugging of Superior Semicircular Canal Dehiscence: Long-Term Follow-Up. Eur. Arch. Oto-Rhino-Laryngol. 2023. Online ahead of print https://doi.org/10.1007/s00405-023-08079-6.
29. Cocca, S.; Mignacco, G.; Mandalà, M.; Giannitto, C.; Esposito, A.A.; Porcino, S. A ‘Double’ Third Window Syndrome: The Case of Semicircular Canal Dehiscence in Twin Sisters. Rep. Med. Imaging 2022, 15, 1–7. https://doi.org/10.2147/RMI.S333347.
30. Dang, P.T.; A Kennedy, T.; Gubbels, S.P. Simultaneous, Unilateral Plugging of Superior and Posterior Semicircular Canal Dehiscences to Treat Debilitating Hyperacusis. J. Laryngol. Otol. 2014, 128, 174–178. https://doi.org/10.1017/S0022215113003605.
31. Kirtane, M.V.; Sharma, A.; Satwalekar, D. Transmastoid Repair of Superior Semicircular Canal Dehiscence. J. Laryngol. Otol. 2009, 123, 356–358. https://doi.org/10.1017/S0022215108002375.
32. Wijaya, C.; Dias, A.; Conlon, B. Superior Semicircular Canal Occlusion-Transmastoid Approach. Int. J. Surg. Case Rep. 2012, 3, 42–44. https://doi.org/10.1016/j.ijscr.2011.09.003.
33. McCall, A.A.; McKenna, M.J.; Merchant, S.N.; Curtin, H.D.; Lee, D.J. Superior Canal Dehiscence Syndrome Associated with the Superior Petrosal Sinus in Pediatric and Adult Patients. Otol. Neurotol. 2011, 32, 1312–1319. https://doi.org/10.1097/MAO.0b013e31822e5b0a.
34. Huedo-Medina, T.B.; Sánchez-Meca, J.; Marín-Martínez, F.; Botella, J. Assessing Heterogeneity in Meta-Analysis: Q Statistic or I 2 Index? Psychol. Methods 2006, 11, 193–206. https://doi.org/10.1037/1082-989X.11.2.193.
35. Crovetto, M.; Areitio, E.; Elexpuru, J.; Aguayo, F. Transmastoid Approach for Resurfacing of Superior Semicircular Canal Dehiscence. Auris Nasus Larynx 2008, 35, 247–249. https://doi.org/10.1016/j.anl.2007.06.010.
36. Gioacchini, F.M.; Alicandri-Ciufelli, M.; Kaleci, S.; Scarpa, A.; Cassandro, E.; Re, M. Outcomes and Complications in Superior Semicircular Canal Dehiscence Surgery: A Systematic Review. Laryngoscope 2016, 126, 1218–1224. https://doi.org/10.1002/lary.25662.
37. Allsopp, T.; Kim, A.H.; Robbins, A.M.; Page, J.C.; Dornhoffer, J.L. Quality of Life Outcomes after Transmastoid Plugging of Superior Semicircular Canal Dehiscence. Am. J. Otolaryngol.—Head Neck Med. Surg. 2020, 41, 102287. https://doi.org/10.1016/j.amjoto.2019.102287.
38. Pletcher, S.D.; Oghalai, J.S.; Reeck, J.B.; Cheung, S.W. Management of Superior Canal Dehiscence Syndrome with Extensive Skull-Base Deficiency. ORL 2005, 67, 192–195. https://doi.org/10.1159/000086663
